# RURAL-URBAN DISPARITY IN BIOMARKER RISKS FOR CARDIOVASCULAR DISEASE AMONG CHINESE MIDDLE-AGED AND OLDER ADULTS

**DOI:** 10.1093/geroni/igz038.973

**Published:** 2019-11-08

**Authors:** Haowei Wang, Jeffrey E Stokes

**Affiliations:** University of Massachusetts Boston, Boston, Massachusetts, United States

## Abstract

Cardiovascular disease (CVD) is a leading cause of adult mortality in China, accounting for 45% of deaths from noncommunicable disease. Moreover, Chinese health status and health services are disproportionately divided between urban and rural areas. This study examined rural-urban differences in age trajectories of CVD risk, measured by C-reactive protein (CRP), high-density lipoprotein (HDL) cholesterol, body mass index (BMI), and waist circumference. This study also investigated whether community factors, including recreational amenities, infrastructure availability, physical environment, public facilities, and health services, may explain such rural-urban disparities. We used data from the baseline data of the China Health and Retirement Longitudinal Study (2011), including 11, 528 respondents from 440 communities, who were aged 45 and older and participated in the biomarker survey. Multilevel models revealed that rural adults had a higher level of HDL and lower levels of CRP, BMI, and waist circumference compared to their urban counterparts. Rural adults also had slower age-related increases in trajectories for CRP, HDL and BMI. Associations of physical environment and public facilities with CVD risks were largely explained by rural-urban disparity. However, the availability of infrastructure explained both between- and within- rural-urban differences in BMI and waist circumference. Models were controlled for previously diagnosed CVD conditions, individual demographic characteristics, self-rated health, activities of daily living, depressive symptoms, physical activity, smoking and drinking behaviors. Findings contribute to the understanding of prevalence and disparities in biomarker risks for CVD among Chinese middle-aged and older adults. Intervention implications are discussed to address the emerging health disparities.

